# Editorial: Next generation nanomaterials for photodynamic therapy

**DOI:** 10.3389/fchem.2022.1123216

**Published:** 2022-12-21

**Authors:** Carlos J. P. Monteiro, Leandro M. O. Lourenço, Heidi Abrahamse, Juan L. Vivero-Escoto, M. Amparo F. Faustino

**Affiliations:** ^1^ LAQV-Requimte and Department of Chemistry, University of Aveiro, Aveiro, Portugal; ^2^ Laser Research Centre, Faculty of Health Sciences, University of Johannesburg, Johannesburg, South Africa; ^3^ Department of Chemistry, University of North Carolina Charlotte, Charlotte, NC, United States

**Keywords:** nanomaterials, nanocarriers, photodynamic therapy, photosensitizers, reactive oxygen species, singlet oxygen

Photodynamic therapy (PDT) is a clinically approved treatment for oncologic and non-oncologic diseases and has several advantages over conventional oncological treatments, such as chemotherapy, radiotherapy, and surgery (Dolmans et al., 2003; Correia et al., 2021). The cytotoxic reactive oxygen species (ROS) produced by the interaction of non-toxic photoactivable molecules (photosensitizers) with dioxygen leads to cancer cell death with a good cosmetic outcome (Pham et al., 2021). Despite the good progress made with photosensitizer (PS)-mediated PDT, this treatment modality has some limitations, such as PS aggregation, low solubility in physiological media, and tissue/cell specificity (Gunaydin et al., 2021).

Recently, several studies based on hybrid nanoparticles were conducted to improve the selectivity and PDT outcome of PSs (Babu et al., 2022). These hybrid materials can circumvent the limitations of conventional PS drugs, increasing their solubility in biological media and improving PS uptake in targets (Lim et al., 2013). Thus, there is significant room for improvement in new generations of nanomaterials and PS agents that can bring about a novel generation of PSs.

The Research Topic ‘Next generation nanomaterials for photodynamic therapy’ comprises five reviews covering recent developments in the fields of nanomaterials for PDT, mechanistic studies, and improving the targeting of nanoplatforms.

Uprety et al. explored the potential of quantum dots (QDs) as PSs for PDT. The versatility of QDs was highlighted, particularly their optical properties, which can be fine-tuned by varying their size. It is worth mentioning that the main advantage of QDs over conventional PSs is the modulation of emission properties within the near IR, consequently allowing deep-light penetration in cancer tissues. Heavy metal QD toxicity was also addressed as a possible drawback for the biological application of QDs; however, solutions for circumventing this issue were disclosed. Reports on the imaging and PDT of cancer using carbon (graphene) and metallic (Cd)-based QDs were also critically reviewed.

George et al. summarized the development, fundamentals, and applications of plasmonic metallic nanoparticles (NPs) for the improvement of PDT approaches. The properties of plasmonic metallic NPs were discussed and the advantages of this treatment modality over conventional PSs was highlighted. The authors paid particular attention to the mechanistic pathways of plasmonic metallic NPs to support enhancement strategies for ^1^O_2_ generation.

State of the art developments in biocompatible nanocarriers and their targeting efficiency in PDT were summarized by Kumar et al. The authors focused particular attention on three types of biocompatible nanoparticles: polymeric nanoparticles, liposomes, and dendrimers. Excellent opportunities in targeted PDT for various types of cancers provided by nanoengineering were reported and corroborated by the obtained results from *in vitro* and *in vivo* studies. Additionally, passive and active targeting mechanisms were discussed and considered when using biocompatible nanocarriers to deliver therapeutic drugs/photosensitizers.

An updated summary of the literature concerning the use of nanoscale metal-organic frameworks (NMOFs) as PSs and nanocarriers for PDT was presented by Matlou et al. in their review paper. The authors presented an introduction to the chemistry/preparation of NMOFs and optimization for their application as PSs in PDT. The NMOFs’ morphology, size, and selectivity for cancer cells over healthy cells were also addressed, taking into consideration the correlation with the outcome efficacy of PDT treatment. Current *in vitro* and *in vivo* studies were also explored and the targeting strategies that improve overall PDT efficacy were pointed out, i.e., subcellular internalization and stimulus-activated release.

Finally, Rezende et al. reviewed the application of rare earths (RE) as upconversion nanomaterials in PDT and bioimaging. They reported the great advantages of using RE elements for PDT applications of nanomaterials due to their optical properties: UV to NIR excitation wavelengths; photoluminescence emissions; long luminescence decay lifetimes (µs to ms); and high sensitivity. Additionally, the authors highlighted the main advantages of REs for diagnostic imaging: the green and NIR emissions can be used to obtain images in diagnostics; moreover, the red emission can contribute to the therapeutic modality. Furthermore, absorption of NIR light is very valuable because it allows greater penetration depth, thus activating the biophotoluminescent material within the cell.

The Editors hope that the contributions collected in this Research Topic will make an impact on the field, bringing new insights into the research, development, and knowledge on the application of nanoparticles in photodynamic therapy.
